# Energy and Nutrients from Apple Waste Using Anaerobic Digestion and Membrane Technology

**DOI:** 10.3390/membranes12090897

**Published:** 2022-09-17

**Authors:** Isabel González-García, Berta Riaño, Beatriz Molinuevo-Salces, María Cruz García-González

**Affiliations:** Agricultural Technological Institute of Castilla y León. Ctra. Burgos, km. 119, 47071 Valladolid, Spain

**Keywords:** nutrient recovery, bioeconomy, waste valorization

## Abstract

The worldwide increment of food waste requires innovative management solutions, aligned with sustainability, energy, and food security. Anaerobic digestion (AD), followed by nutrient recovery, may be considered an interesting approach. This study proposed a co-digestion of apple pomace (AP) with swine manure (SM) to study the effect of different proportions of AP (0, 7.5, 15, and 30%, on a volatile solids (VS) basis) on the methane production and the stability of the process. Subsequently, the gas-permeable membrane (GPM) technology was applied to recover nitrogen (N) as ammonium sulfate (bio-based fertilizer) from the digestates produced after the AD of 7.5% of AP and SM, and SM alone. The results showed that the co-digestion of 7.5% and 15% of AP with SM presented a methane production similar to the AD of SM alone (with 412.3 ± 62.6, 381.8 ± 134.1, and 421.7 ± 153.6 mL g VS^−1^ day^−1^, respectively). The later application of the GPM technology on the resulting digestates, with SM alone and with 7.5% of AP with SM, showed total ammoniacal N recovery rates of 33 and 25.8 g N m^−2^ d^−1^, respectively. Therefore, the AP valorization through the AD process, followed by N recovery from the digestate, could be a good management strategy.

## 1. Introduction

The apple production in the world in 2020 was 86.44 million tons, of which 11.48 million tons came from the European Union [1]. Around 20% of this production is used by the food industry to produce cider, jelly, and juice as the main products [2]. After this processing, 25% of the fresh apple becomes waste, in the form of skin, seeds, and pulp, known as apple pomace (AP), which is usually destined for landfills, incineration, or composting [2]. These forms of disposal generate environmental and human health issues related to greenhouse gas emissions [2,3].

In recent years, the valorization of agro-industrial and food waste through its transformation into by-products with added value has gained importance as one of the main solutions for waste management, following the principle of the end-of-waste and nutrient recovery criteria within the circular economy model [4]. One of the main technologies for waste management is anaerobic digestion (AD), a consolidated process widely used for livestock waste treatment [5,6]. AD allows for the production of biogas from agriculture waste and residues, such as manure, contributing to the production of renewable energy, while the resulting effluent of the process (digestate) can be used as a valuable organic fertilizer, reducing the dependency on expensive mineral fertilizers [7]. Several works have studied the co-digestion of different food wastes and manure over the last few years. For example, Labatut et al. [8] co-digested different co-substrates (cheese whey, plain pasta, used vegetable oil, cabbage, and raw potatoes, among others) with dairy manure. Bres et al. [9] studied the performance of semi-continuous AD by co-digesting poultry manure with fruit and vegetable waste, while Riaño et al. [10] studied the co-digestion of pepper waste and swine manure (SM). 

However, only a few works have studied the use of apple residues as co-substrates for the AD process, and most of these studied the process under batch conditions. 

Riggio et al. [11] studied apple waste valorization using different mixtures of apple pulp, cow manure, and olive pomace and compared the methane production under batch conditions, obtaining the highest methane production (216.3 L CH_4_ kg volatile solids (VS)^−1^) with a mixture of 85% cattle slurry, 10% olive pomace, and 5% apple pulp (*w*/*w*). Molinuevo-Salces et al. [12] studied the effect of AP in the mixture during the co-digestion with SM in the range of 0–100% (on a VS basis) following a central composite design, obtaining the highest methane yield (596 mL CH_4_ gVS^−1^) with 14.6% of AP in the mix. They also found differences between the methane potential of apple residues from the cider industry and the juice industry. Kafle and Kim [13] studied the co-digestion of fresh crushed apples as apple waste (AW) and SM, using different proportions of AW in the mixture under semi-continuous conditions (AW:SM ratios of 25:75; 33:67, and 50:50 on a VS basis). They observed positive synergetic effects on biogas production when the AW content in the feed increased from 25% to 33% (on a VS basis), but a further increase in the AW content from 33% to 50% had a negative synergetic effect due to the rapid accumulation of total volatile fatty acids (TVFA). Nevertheless, as the substrate used by these authors was fresh apples instead of real waste, it may not perform similarly to industrial apple waste. Therefore, a deeper knowledge of the use of real apple waste as a substrate in anaerobic co-digestion with SM under semi-continuous regimen is required. Furthermore, the study of the potential relationship between AD and nutrient recovery has arisen as an important new trend [5]. 

The use of the digestate as organic fertilizer may involve storage and transportation problems, as well as land application limitations related to the addition of nitrogen (N) rich substances in agricultural fields, leading to nitrate emissions and nutrient losses [14,15]. The application of nutrient recovery technologies can address these issues and prevent nutrient losses; thus, the interest and incentives for nutrient recovery and reuse of AD effluents have been increasing over the last few years, [4,16]. For N recovery from a digestate, the most commonly used technologies are air stripping, ion exchange, ultrafiltration, reverse osmosis, or gas-permeable membrane technology (GPM) [17]. Specifically, the GPM technology is considered as one of the most valuable novel technologies to recover N, attending to both economic and energetic aspects [17,18,19]. This technology is based on the mass transfer driven by the difference in ammonia (NH_3_) gas concentration between both sides of a gas-permeable hydrophobic membrane. The membrane is submerged in the wastewater, and the NH_3_ passes through the pores. Then it is trapped by the acidic solution that circulates inside the membrane, being recovered as an ammonium salt solution (bio-based fertilizer) [20,21]. In this way, the treated digestate can be applied in the field to provide the advantages of a fertilizer rich in organic matter, but without N excess. The GPM technology has been successfully used in several studies for N recovery from such organic wastes as SM, obtaining N recovery percentages up to 98% [22,23,24], and from different digestates generated in anaerobic co-digesting systems, with N recovery percentages up to 99% [6,25,26]. Some of these studies used SM co-digested with organic co-substrates, such as fruit and vegetable sludge from peppers and artichokes, as well as by-products of the tomato processing industry [26], or tobacco powder and cereal powder [6]. However, so far, the N recovery from digestates of AP residues has not been addressed. 

To the best of our knowledge, a study of the efficiency of the AD process with different proportions of AP as the co-substrate, with the subsequent recovery of N from the resulting digestate, has never before been conducted. The aim of the present work was to assess the effect of the addition of different ratios of AP from the cider industry with SM in the AD process, under semi-continuous operation and mesophilic conditions, followed by the application of the GPM technology for the N recovery from the digestate in the form of a valuable ammonium salt solution, and to determine if the use of AP as a co-substrate could affect the N recovery process. This approach can provide valuable information to stakeholders in the agri-food sector, offering a point of view not only focused on energy, but also on the potential production of a bio-based fertilizer.

## 2. Materials and Methods

### 2.1. Origin and Characterization of Apple Pomace, Manure, and Inoculum

AP was provided by the Regional Research and Development Service of Asturias (SERIDA) (Asturias, Spain). The AP was a solid fresh waste obtained after apple pressing for cider production. It was transported to the ITACyL in plastic containers and kept frozen for further use. The AP presented a content of total solids (TS) of 268.7 ± 14.5 g kg^−1^ and a content of VS of 265.4 ± 14.4 g kg^−1^. 

The SM used was centrate collected after on-farm centrifugation from a pig farm located in Narros de Cuéllar (Segovia, Spain). The collected manure was put in plastic containers, transported to the ITACyL laboratory in Valladolid (Spain), and stored in the laboratory at 4 °C for further use. The mean characteristics of the SM were pH of 7.2 ± 0.1, 41.8 ±7.1 g TS L^−1^, 30.6 ± 4.5 g VS L^−1^, 139.1 ± 94.9 g total chemical oxygen demand (TCOD) L^−1^, 36.2 ± 2.2 g soluble chemical oxygen demand (SCOD) L^−1^, 4762 ± 62 mg total Kjeldahl nitrogen (TKN) L^−1^, and 3607 ± 308 mg total ammoniacal nitrogen (TAN) L^−1^.

The inoculum used was obtained from an anaerobic digester of the municipal wastewater treatment plant in Valladolid (Spain). The inoculum had a concentration of 10.9 ± 0.1 g VS L^−1^.

### 2.2. Experimental Set-Up

#### 2.2.1. Semi-Continuous Co-Digestion of Different Mixtures

**Figure 1:**
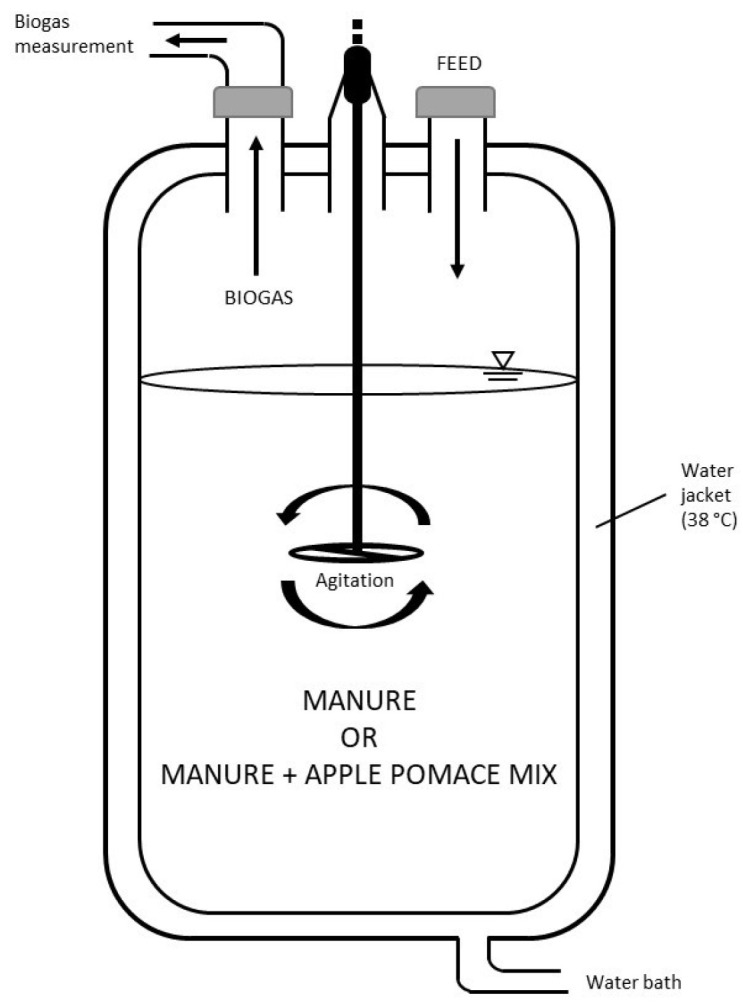
Experimental set-up of an anerobic reactor for the AD experiments. The arrows indicate the direction of the flows.

Semi-continuous co-digestion was carried out using two identical continuously stirred tank reactors (CSTRs) with a total volume of 7 L and a working volume of 5 L, namely R1 and R2. A water bath was used to maintain the temperature of the reactors at 38 °C (mesophilic conditions). The reactors were mounted separately, and the mixture was homogenized by continuous stirring (37 rpm). The outlets provided on the top of each reactor were used for feeding the influent, withdrawing the effluent, and for collecting the biogas. The volume of biogas produced was measured daily by water displacement (Figure 1).

**Figure 2:**
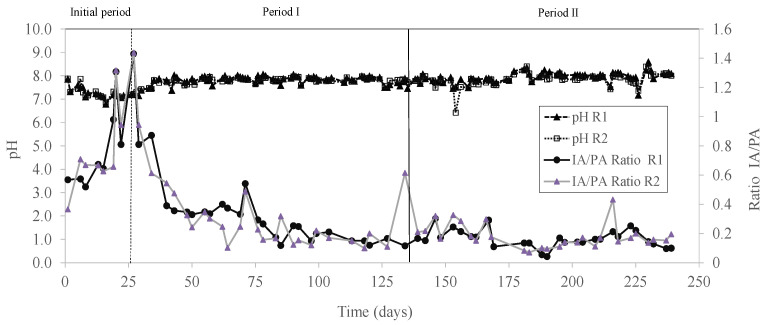
The pH and IA/PA ratio evolution for both reactors during the semi-continuous AD operation.

The semi-continuous co-digestion was performed for 240 days. The reactors were initially filled with 5 L of inoculum. After that, manual feeding of the reactors was performed once per day, every weekday. Prior to each feeding, a volume equal to the feeding volume was removed to maintain a constant reactor volume. Initially, an organic loading rate (OLR) of 1.04 g VS L^−1^ d^−1^ and a hydraulic retention time (HRT) of 25 days were applied in both reactors (initial period). In this initial period (0–26 days), R1 was fed with SM alone; whereas R2 was fed with a mixture of SM and AP in a feed VS ratio of 85.0% SM and 15.0% AP. This percentage was used based on a previous work that investigated the influence of the percentage of AP on anaerobic co-digestion of AP and SM, following a central composite design, and obtaining the highest methane yield with a percentage of AP in the mixture of 15% [12]. Since a progressive destabilization of both reactors occurred during the initial period (Figure 2), the applied OLR was decreased to 0.78 g VS L^−1^ d^−1^, and the HRT was increased to 33 days, maintaining these conditions during the rest of the experiment. 

In period I (27–138 days), R1 was used to digest SM alone; whereas R2 was used to co-digest SM with AP in a feed VS ratio of 85.0% SM and 15.0% AP. In period II (139–240 days), R1 was fed with a mixture of SM and AP in a feed VS ratio of 92.5% SM and 7.5% AP, and R2 was fed with a mixture composed of 70.0% SM and 30.0% AP on a VS basis. Table 1 shows the chemical composition of the influents for R1 and R2 for both periods. 

Influent and effluent samples were taken twice a week and analyzed for total alkalinity (TA), partial alkalinity (PA), TS, VS, TCOD, SCOD, TKN, and TAN. The pH was monitored daily in both the influents and effluents. Total TVFA were obtained from samples every two weeks. The composition of the biogas was analyzed once a week. The methane volumes were converted to standard temperature and pressure (0 °C and 101.325 kPa); therefore, the specific methane yield was calculated as normalized mL of CH_4_ produced per g VS added to the digester per day (N mLCH_4_ VS^−1^). The results were used to evaluate the effect of co-digestion on the biogas production, the process stability, and the biodegradability.

#### 2.2.2. Nitrogen Recovery from Digestate Using Gas-Permeable Membranes

The performance of GPM technology for TAN recovery from the digestates obtained in the experiments described in Section 2.2.1 was evaluated. Therefore, two experiments were carried out using the digestate generated in period I in R1 and R2, namely D-R1 (anaerobically digested SM alone) and D-R2 (anaerobically digested SM and AP, with a ratio of 85% and 15% on a VS basis, respectively). D-R1 was selected as the reference (SM) for the N capture, and D-R2 was selected as the digestate with the highest AP ratio in the feed mix with SM, presenting the best performance in the co-digestion in terms of stability and methane production. This allowed for the discovery of whether the addition of the AP in the co-digestion of SM could influence the further N recovery process. 

**Figure 3:**
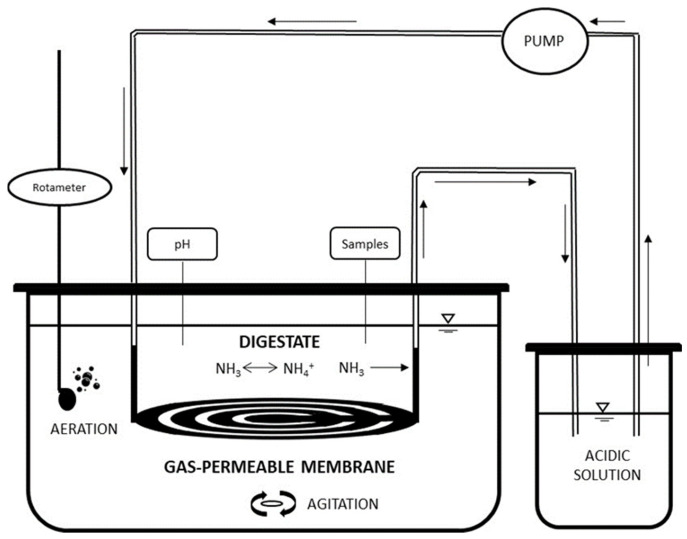
Experimental set-up of the N recovery experiments using the gas-permeable membrane technology. The arrows indicate the direction of the flows. The pH measurement and sampling were carried out through the reactor lid.

Each experiment was performed in duplicate using 2 identical PVC reactors, with a total effective volume of 1.5 L (diameter 16.5 cm, height 17.5 cm), Figure 3.

The acid tanks used to recover TAN consisted of 0.5 L flasks, initially containing 0.3 L of acidic solution (0.5 mol L^−1^ H_2_SO_4_). This acidic solution was continuously recirculated using a peristaltic pump (Pumpdrive 5001, Heildolph, Schwabach, Germany) through a gas-permeable tubular membrane. The recirculation flow rate of the acidic solution through the tubular membrane was 9 L d^−1^. The tubular membrane was made of expanded polytetrafluoroethylene (e-PTFE) (Zeus Industrial Products Inc., Orangeburg, SC, USA), with a length of 120 cm, an external diameter of 5.2 mm, a wall thickness of 0.56 mm, and a density of 0.95 g cm^−3^. The ratio of the membrane surface area to the volume of the digestate was 0.013 m^2^ L^−1^. The tubular membrane was placed in a horizontal configuration and held by plastic connectors to ensure that the membrane was completely submerged throughout the experiments. To keep the pH of the digestate high and thus ensure the formation of NH_3_ [22], air was supplied using an aquarium pump (Hailea, Aco-2201, Guangdong, China) from the base of the reactor through a porous stone. The air flow rate was controlled by a rotameter (Aalborg, Orangeburg, NY, USA) and was kept constant at 0.24 L air L^−1^ min^−1^. The reactors were not hermetically sealed. The pH of the acidic solution was kept under 2 to optimize the TAN recovery process. Thus, the pH of the acidic solution was monitored daily, and whenever the pH reached 2, concentrated H_2_SO_4_ (96–98%, Panreac) was added to an endpoint of pH <2. All experiments were carried out at room temperature (25 ± 1 °C). The duration of the experiments was 7 days. Daily samples of the digestate and acidic solution were taken for pH and TAN determination. Analyses of the TS, VS, TA, and TKN of the digestates were also performed at the beginning and end of the experiments. 

The TAN removal, TAN recovery efficiencies, and average TAN recovery rate by the GPM system in the digestate were calculated according to Equations (1)–(3), respectively:

(1) $$TAN\;removal\;\left(\%\right)=\frac{\left(Initial\;mass\;of\;TAN-Final\;mass\;of\;TAN\right)}{Initial\;mass\;of\;TAN}\;*100$$

(2) $$TAN\;recovery\;\left(\%\right)=\frac{Final\;mass\;of\;TAN\;in\;the\;acidic\;solution}{mass\;of\;TAN\;removed\;from\;the\;digestate}*100$$

(3) $$Average\;TAN\;recovery\;\left(mg\;N\;m^{-2}\;day^{-1}\right)=\frac{\begin{array}{c} mass\;of\;TAN\;recovered\;\\ in\;the\;acidic\;solution \end{array}}{\begin{array}{c} m^{2}\;of\;membrane\;and\;\\ \begin{array}{c} days\;of\;experiment\; \end{array} \end{array}}*100$$

Free ammonia (FA) was calculated using the equation of Hansen et al. [27], as un-ionized ammonia (Equation (4)):

(4) $$\frac{{\mathrm{NH}}_{3}^{}}{{\mathrm{tNH}}_{3}^{}}=(1+{\left(10^{-pH}/10^{-\left(0.09018+2729.92/T\right)}\right)}^{-1}$$

where NH_3_ was the FA content, tNH_3_ was the total NH_3_ concentration, and the pH and T (in Kelvin) were measured in the digestate inside the reactors.

### 2.3. Analytical Methods and Statistical Analysis

Analyses of TCOD, SCOD, TS, VS, TKN, and TAN were performed in duplicate in accordance with APHA [28]. For the analysis of TCOD and SCOD, a closed reflux colorimetric method was used. TS content was calculated by drying the sample to a constant weight at 103–105 °C. The TS residue was ignited at 550 °C to constant weight, and therefore, the weight lost on ignition corresponded to the VS content. The TKN was measured using the Kjeldahl digestion, distillation, and titration method. TAN was measured using the distillation and titration method. The pH was measured using a Crison Basic 20 pH-meter (Crison Instruments S.A., Barcelona, Spain). Total and partial alkalinity (TA, PA) were calculated by measuring the amount of 0.1 N H_2_SO_4_ needed to bring the sample to a pH of 4.3 and 5.75, respectively, and expressed as mg CaCO_3_ L^−1^. Intermediate alkalinity (IA) was calculated by subtracting the PA from TA. 

The TVFA concentrations (acetic, propionic, hexanoic, hepta, isobutyric, butyric, isovaleric, valeric, hexanoic, and heptanoic) were determined using a gas chromatograph (Agilent 7890A, Santa Clara, CA, USA) with a Teknokroma TRB-FFAP column of 30 m length and 0.25 mm diameter, immediately followed by a flame ionization detector (FID), where the carrier gas was helium (1 mL min^−1^) and the temperature of the detector and the injector was 280 °C. TVFA were calculated as the sum of those acid concentrations after applying the corresponding TCOD conversion factor.

Biogas composition was determined using a gas chromatograph (Agilent 7890A, Santa Clara, CA, USA) with a thermal conductivity detector, provided by an HP-Plot column (30 m 0.53 mm 40 μm), followed by an HP-Molesieve column (30 m 0.53 mm 50 μm). The carrier gas was helium (7 mL min^−1^). The injection port temperature was set at 250 °C, and the detector temperature was 200 °C. The temperature of the oven was set at 40 °C for 4 min and was thereafter increased to 115 °C. 

The results obtained were analyzed using one-way ANOVA, with significance at *p* < 0.05.

## 3. Results and Discussion

### 3.1. Semi-Continuous Co-Digestion of Different Mixtures

**Figure 4:**
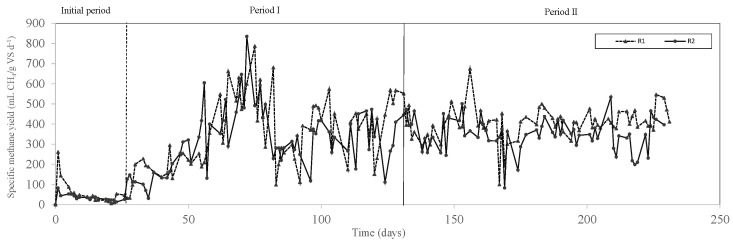
Methane yields during semi-continuous operation of R1 and R2.

During the initial period, an OLR of 1.04 g VS L^−1^ d ^−1^ and an HRT of 25 days were applied. As can be seen from Figure 4, a decrease in the specific methane yields in both reactors was observed from day 6.

In addition, an increase in the IA/PA ratios was also detected from day 6 (Figure 2). The ratio IA/PA plays a functional role in providing a rapid response to unstable states of the anaerobic digestion process. Thus, an IA/PA ratio of below 0.3 indicates a good performance of the anaerobic process [29]. In this study, the high IA/PA ratios (from 0.36 up to 1.4) during the initial period, along with the low methane yields, indicated an unstable operation of the anaerobic process under the two studied conditions (i.e., SM alone in R1, and co-digestion of SM and AP in a feed ratio of 15% on a VS basis in R2). The instability could be caused by a short HRT, since feeds containing fiber or celluloses (as is the case with the AP) may require relatively longer HRT [30]. For this reason, the OLR applied in both reactors was decreased to 0.78 g VS L^−1^ d^−1^, and the HRT was increased to 33 days in periods I and II.

The values of the monitored parameters in both reactors during periods I and II are summarized in Table 2.

Similar specific methane yields (*p* > 0.05) were obtained when digesting SM alone and with up to 15% of AP in the feed mixture, with average values varying between 421.7 N mL CH_4_ g VS^−1^ d^−1^ and 381.8 N mL CH_4_ g VS^−1^ d^−1^ (Table 2; Figure 4). However, a significant decrease in the specific methane yield (i.e., 341.9 N mL CH_4_ g VS^−1^ d^−1^) was detected when digesting a feed mixture with 30% of AP in R2 in period II (Table 2). Similarly, the methane content significantly decreased from 61.6% to 58.0% when increasing the AP in the feed to 30% (Table 2). In the present study, the pH in both reactors was constant during the whole experimental period, approximately in the range of 6.8 and 8.6 (Figure 2). Additionally, the IA/PA ratios were lower than 0.3 (Table 2; Figure 2), which indicated that the reactors were stable during both experimental periods. The TVFA concentration in R1 in period I was 5755 ± 18 mg COD L^−1^, and it decreased to 1767 ± 586 mg COD L^−1^ in period II (Table 2). In the same line, the TVFA concentration in R2 in period I was 1661 ± 119 mg COD L^−1^, and it decreased to 144 ± 9 mg TCOD L^−1^ in period II (Table 2). Therefore, a VFA accumulation did not occur.

In the case of Kafle and Kim [13], the destabilization and the decrease in the methane production of the AD process occurred due to the accumulation of VFA when the AW of the mix was increased to 50% (on VS basis). In this study, the decrease in the specific methane yield with the increment of AP in the feed mix up to 30% (on VS basis) might be caused by the lower biodegradability of the AP in comparison with the SM. Labatut et al. [8] stated that co-digestion with lignocellulosic substrates could imply lower biodegradability, therefore leading to lower methane yields. AP presents a high fiber (or lignocellulose) content, which is composed of cellulose, hemicellulose, and lignin, all of which are poorly biodegradable compounds. The fiber content of AP could be between 51–58% (on a dry weight basis), with 15–24% lignin, 12% hemicellulose, and 7–31% cellulose [31,32]. The AP used in this study contained 16.1% hemicellulose and 21.5% lignin (on TS) [12]. Hence, the increase in the AP percentage in the feed mix up to 30% would imply a significant increase in the lignocellulose in the substrate, hindering the biodegradability and leading to a significantly lower methane production.

There were no significative differences in the TCOD removal efficiencies, regardless of the percentage of AP in the mix (Table 2). The TCOD is a measurement of the chemically oxidizable material, so it indicates the maximum chemical energy present in the material [33]. Thus, similar TCOD removal efficiencies in both reactors in all periods showed that the chemically oxidizable components did not change significantly during the experiment. Similarly, the VS removal efficiency ranged between 31.1% and 44.3%, with no significant differences when comparing the digestion of SM alone and the co-digestion of SM and 30% AP (Table 2). However, the SCOD removal efficiency increased with the increment in the proportion of AP in the fed mix (Table 2). Although the SCOD concentrations of the influent were similar in all periods in both reactors, with values varying between 32.3 and 37.8 g L^−1^ (Table 1), the different nature of the organic compounds and their biodegradability could lead to different methane productions.

The results showed that the methanogenic potential of fresh solid AP residues from the cider industry can be improved by the co-digestion of the AP with SM. The AD process in the reactors, where AP was used as the feed, had a similar AD performance to the one with SM alone, and a decrease in the specific methane yield was only observed when the AP content was increased to 30% in the feed mix (on a VS basis). Thus, despite the fact that no improvement in the methane production was detected with the addition of AP, the AP can be used as an AD substrate, thus relieving the industries in the area of the burden of this residue. With an adequate proportion, the reactor can operate with high biogas yields, without collapsing due to the accumulation of volatile fatty acids associated with the organic fraction of the substrate.

### 3.2. Nitrogen Recovery with GPM Technology

For the N recovery experiments, two different digestates were used: D-R1 (anaerobically digested SM with no addition of AP), and D-R2 (anaerobically digested SM with 15% of AP in the feed mixture). DR-1 and D-R2 presented different initial concentrations of TKN (4650 and 3300 mg N L^−1^, respectively), TAN (3845 and 2944 mg N L^−1^, respectively), VS (20.43 and 12.89 g L^−1^, respectively), alkalinity (20115 and 15,668 mg CaCO_3_ L^−1^, respectively) and pH (8.57 and 8.20, respectively). 

**Figure 5:**
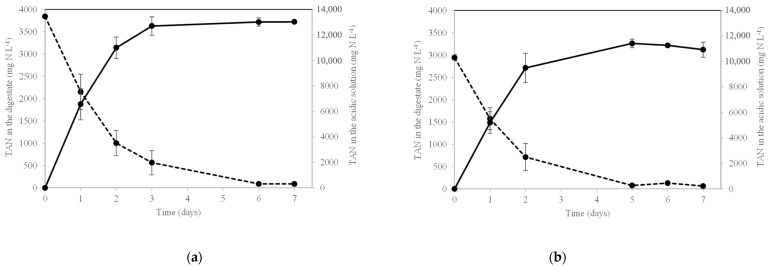
Evolution of TAN concentration in the digestates in D-R1 (**a**), and D-R2 (**b**) (discontinuous line), and TAN concentration in the acidic solutions for D-R1 (**a**) and D-R2 (**b**) (continuous line).

For DR-1, the TAN concentration in the digestate decreased to 89.4 mg N L^−1^ on day 7 (Figure 5a), leading to a TAN removal efficiency of 97.6% (Table 3).

For DR-2, the TAN concentration decreased to 65 mg N L^−1^ on day 7 (Figure 5b), with a TAN removal efficiency of 97.8% (Table 3).

The percentage of removed TAN that was recovered by the GPM in the form of ammonium salt was 77.2% for D-R1 and 75.8% for D-R2 (Table 3). The TAN concentration in the acidic solutions on day 7 reached values of 13,029 ± 106 mg N L^−1^ for DR-1 and 10,924 ± 596 mg N L^−1^ for D-R2 (Figure 5a,b). 

**Figure 6:**
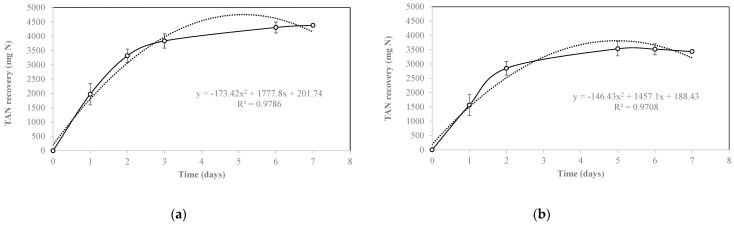
TAN recovery in the acidic solutions of DR1 (**a**) and D-R2 (**b**). Continuous lines correspond to TAN recovery, and dotted lines correspond to second order polynomial curves.

For both D-R1 and D-R2, most of the TAN recovery occurred in the first 4 days of the experiment. In both cases, the TAN recovery was much faster in the early days, as it followed a second order equation (Figure 6). 

The decrease in the TAN recovery rate can be attributed to the reduction in the FA content in the digestate over time. In this context, the FA content in wastewater has been stated as one of the main variables affecting TAN recovery using the GPM technology, along with pH and temperature. The pH affects the acid-based chemical balance of ammonium/ammonia (NH_4_^+^/NH_3_) in the wastewater, where values near 9 cause the release of ammonia, passing thorough the membrane pores to be captured by the acid [23] (Figure 6). 

**Figure 7:**
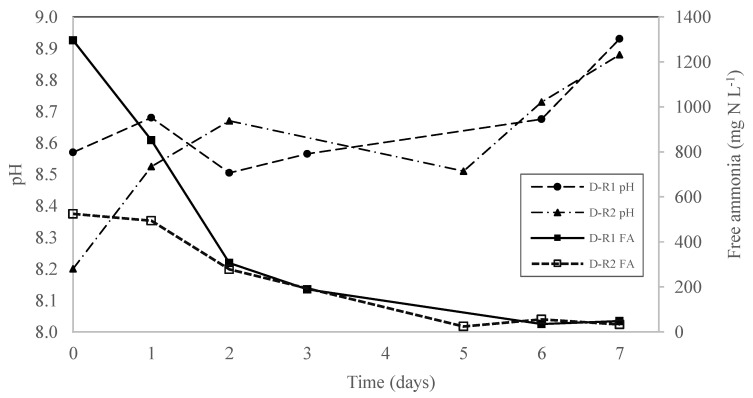
The evolution of the pH and the free ammonia (FA) for D-R1 and D-R2 during the N recovery experiments.

In the present study, a clear reduction in the TAN recovery was found from day 2 for both digestates, in which the FA content was 305 mg N L^−1^ for D-R1 and 278 mg N L^−1^ for DR-2 (Figure 7). 

These values were higher than the thresholds reported in the literature. For example, Riaño et al. [10] observed that the TAN recovery rate decreased when the FA content in the digestate was lower than 95 mg N L^−1^. The average TAN recovery rate was 22% lower for D-R2 (25.8 g N m^−2^ day^−1^) than for D-R1 (32.9 g N m^−2^ day^−1^) (Table 3). This could be attributed to the higher FA content availability in D-R1 as compared to D-R2, as a consequence of its higher initial TAN concentrations and similar values of pH throughout the experimental period (Figure 7). In this vein, García-González and Vanotti [22] studied TAN recovery from swine manure with different TAN concentrations and concluded that, as available FA content increased in the manure, the TAN recovery rate also increased. Therefore, although the co-digestion of AP and SM did not affect the subsequent N recovery using GPM technology, it did affect the TAN recovery rates, which should be considered in larger scale nutrient recovery processes. 

The application of this type of innovative solution favors energy and agronomic autonomy, while representing a solution for the treatment of waste from the agri-food industry, according to the principles of the circular bio-economy.

## 4. Conclusions

The results showed that fresh AP from the cider industry can be successfully valorized through anaerobic digestion. With up to 15% of AP in the feed mix with SM (on a VS basis), a methane yield of 381.8 ± 134.1 mL CH_4_ g ^−1^ VS day ^−1^ was achieved, while higher percentages may decrease the methane yields. The sequential treatment of the AD digestate using the novel GPM technology enables an up to 76% recovery of the N present in the form of an ammonium sulfate solution, with a concentration of 10,924 ± 596 mg N L^−1^. This solution is a bio-based fertilizer which can be easily stored, transported, and directly applied to crops, without any necessary post-treatment, avoiding the use of mineral fertilizers. Therefore, this organic waste treatment combination of anaerobic digestion and membrane technology can be considered as an efficient form of waste valorization for energy and nutrient generation.

## Figures and Tables

**Table 1:** Composition of feedstocks at different mixture ratios. Standard deviations are shown in parenthesis. No significant differences were found among the analytical values (*p* > 0.05).

	R1			R2		
Parameters	Initial Period	Period I	Period II	Initial Period	Period I	Period II
Apple pomace (%)	0	0	7.5	15	15	30
pH	7.19 (0.14)	7.40 (0.36)	6.98 (0.24)	7.19 (0.14)	7.40 (0.36)	6.98 (0.24)
TA (mg CaCO_3_ L^−1^)	11882 (1565)	13626 (3268)	8453 (2007)	11882 (1565)	13626 (3268)	8453 (2007)
TCOD(g L ^−1^)	107.11 (60.54)	92.00 (23.43)	99.90 (25.23)	89.58 (60.18)	81.06 (17.32)	90.09 (16.60)
SCOD (g L ^−1^)	32.51 (5.27)	36.39 (4.81)	37.81 (1.79)	31.77 (3.42)	32.26 (6.91)	35.92 (6.77)
TS (g L ^−1^)	40.51 (0.41)	42.79 (10.98)	50.99 (12.69)	43.68 (2.03)	38.43 (11.34)	44.95 (9.57)
VS (g L ^−1^)	28.95 (4.11)	24.83 (6.53)	36.74 (12.86)	29.15 (1.92)	21.91 (5.49)	32.68 (9.75)
TVFA(mg TCOD L^−1^)	n.d.	26243 (5839)	31883 (1421)	n.d.	19219 (394)	26735 (6218)
TKN (mg N L ^−1^)	5121 (391)	5085 (823)	5048 (670)	4665 (289)	4519 (465)	4235 (628)
TAN (mg N L ^−1^)	3806 (179)	3793 (193)	3874 (424)	3286 (118)	3325 (221)	3182 (480)

n.d.: not determined. TA stands for total alkalinity; TCOD stands for total chemical oxygen demand; SCOD stands for soluble chemical oxygen demand; TS stands for total solids; VS stands for volatile solids; TVFA stands for total volatile fat acids; TKN stands for total Kjeldahl nitrogen; TAN stands for total ammoniacal nitrogen.

**Table 2:** Performance of AD treatment of four mixtures of SM and AP. Standard deviations are shown in parenthesis, followed by the descriptive coefficients indicating the statistically significant differences. The means within each parameter (horizontal) followed by different letters represent significant differences (*p* < 0.05).

	R1		R2	
Parameters	Period I	Period II	Period I	Period II
Apple pomace (%)	0.0	7.5	15.0	30.0
Biogas (mL day^−1^)	3057 (1255) ^a^	3529 (542) ^b^	2828 (1135) ^a^	3030 (1130) ^a^
Methane (CH_4_) (%)	61.57 (4.28) ^a^	61.96 (1.23) ^a^	59.28 (2.04) ^ab^	57.96 (3.02) ^b^
Specific methane yield (mL g VS^−1^ day ^−1^)	421.7 (153.6) ^a^	412.3 (62.6) ^a^	381.8 (134.1) ^ab^	341.9 (78.1) ^b^
TCOD reduction (%)	52.78 (13.43) ^a^	51.27 (11.50) ^a^	57.01 (12.07) ^a^	54.85 (15.10) ^a^
SCOD reduction (%)	48.57 (14.42) ^a^	58.94 (11.53) ^b^	67.13 (7.13) ^cd^	70.58 (7.79) ^d^
VS reduction (%)	32.29 (15.01) ^a^	44.27 (15.98) ^b^	31.05 (10.48) ^ab^	39.71 (14.51) ^ab^
TVFA (mg TCOD L^−1^)	5755 (18) ^a^	1767 (586) ^b^	1661 (119) ^b^	144 (9) ^c^
IA/PA ratio	0.23 (0.11) ^a^	0.15 (0.05) ^b^	0.22 (0.14) ^b^	0.25 (0.07) ^b^

TCOD stands for total chemical oxygen demand; SCOD stands for soluble chemical oxygen demand; VS stands for volatile solids; TVFA stands for total volatile fat acids; IA/PA ratio stands for intermediate alkalinity/partial alkalinity ratio.

**Table 3:** Mass balances of the recovery of TAN from the digestates D-R1 and D-R2.

		TAN in Manure (mg N)	TAN in the Acidic Solution (mg N)	TAN Removal Efficiency (%)	TAN Recovery Efficiency (%)	Average TAN Recovery Rate (g N m^−2^ day^−1^)
D-R1	Initial	5768 (0)	0 (0)	97.6	77.2	32.9
	Final	133 (44)	4383 (42)			
D-R2	Initial	4416 (73)	0 (0)	97.8	75.8	25.8
	Final	98 (17)	3434 (136)			

TAN stands for total ammoniacal nitrogen.

## Data Availability

The data that support the findings of this study are available from the corresponding author (ITACyL, Agricultural Technological Institute of Castilla y León. Ctra. Burgos, km. 119, 47071 Valladolid, Spain) upon request.
